# Proposing Metrics for Benchmarking Novel EEG Technologies Towards Real-World Measurements

**DOI:** 10.3389/fnhum.2016.00188

**Published:** 2016-05-10

**Authors:** Anderson S. Oliveira, Bryan R. Schlink, W. David Hairston, Peter König, Daniel P. Ferris

**Affiliations:** ^1^Human Neuromechanics Laboratory, School of Kinesiology, University of MichiganAnn Arbor, MI, USA; ^2^Translational Neuroscience, Human Research and Engineering Directorate, U.S. Army Research LaboratoryAdelphi, MD, USA; ^3^Institute of Cognitive Science, University of OsnabrückOsnabrück, Germany; ^4^Department of Neurophysiology and Pathophysiology, University Medical Center Hamburg-EppendorfHamburg, Germany

**Keywords:** EEG, event-related potentials, walking, locomotion, reliability, mobility, validation metrics

## Abstract

Recent advances in electroencephalographic (EEG) acquisition allow for recordings using wet and dry sensors during whole-body motion. The large variety of commercially available EEG systems contrasts with the lack of established methods for objectively describing their performance during whole-body motion. Therefore, the aim of this study was to introduce methods for benchmarking the suitability of new EEG technologies for that context. Subjects performed an auditory oddball task using three different EEG systems (Biosemi wet—BSM, Cognionics Wet—Cwet, Conionics Dry—Cdry). Nine subjects performed the oddball task while seated and walking on a treadmill. We calculated EEG epoch rejection rate, pre-stimulus noise (PSN), signal-to-noise ratio (SNR) and EEG amplitude variance across the P300 event window (CV_ERP_) from a subset of 12 channels common to all systems. We also calculated test-retest reliability and the subject’s level of comfort while using each system. Our results showed that using the traditional 75 μV rejection threshold BSM and Cwet epoch rejection rates are ~25% and ~47% in the seated and walking conditions respectively. However, this threshold rejects ~63% of epochs for Cdry in the seated condition and excludes 100% of epochs for the majority of subjects during walking. BSM showed predominantly no statistical differences between seated and walking condition for all metrics, whereas Cwet showed increases in PSN and CV_ERP_, as well as reduced SNR in the walking condition. Data quality from Cdry in seated conditions were predominantly inferior in comparison to the wet systems. Test-retest reliability was mostly moderate/good for these variables, especially in seated conditions. In addition, subjects felt less discomfort and were motivated for longer recording periods while using wet EEG systems in comparison to the dry system. The proposed method was successful in identifying differences across systems that are mostly caused by motion-related artifacts and usability issues. We conclude that the extraction of the selected metrics from an auditory oddball paradigm may be used as a benchmark method for testing the performance of different EEG systems in mobile conditions. Moreover dry EEG systems may need substantial improvements to meet the quality standards of wet electrodes.

## Introduction

Descriptions of brain activity using non-invasive electroencephalographic (EEG) have become an important topic in recent years. Special attention has been devoted to establish methodologies for successfully recording brain activity in real-world conditions (Casson et al., 2010; McDowell et al., 2013; Ries et al., 2014; Mihajlovic et al., 2015; Oliveira et al., 2016). The popular framework of embodied cognition requires investigating and modeling natural brain dynamics (Gramann et al., 2014). Along these lines, investigating sensorimotor coupling requires allowing a wide range of motor actions like complex movements, locomotion and navigation (Gwin et al., 2010; Ehinger et al., 2014). However, due to the high dynamic range and low signal-to-noise in these types of situations, acquiring reliable records of brain electrical activity in these motion conditions requires robust EEG acquisition systems beyond what has been conventionally used in more restricted laboratory or medical scenarios. Such achievement can contribute substantially to advance many research areas, such as psychology, motor rehabilitation, aging, robotics, and user-system interaction (Lance et al., 2012; McDowell et al., 2013; Kranczioch et al., 2014).

In the past few years, new EEG data acquisition approaches have been developed which leverage wireless data transmission and wet or dry electrodes as an attempt to increase the general usability of the systems for non-traditional use (Chi et al., 2010; Grozea et al., 2011; Zander et al., 2011; Hairston et al., 2014; Lopez-Gordo et al., 2014). While promising, an ongoing challenge is how to validate or prove the overall efficacy of these approaches, since there currently are no community-accepted or standard metrics for EEG signal quality as observed within real-world domains. In most cases, authors use the output from classic tasks such as steady-state visual evoked potential and oddball paradigms in stationary conditions as an attempt to benchmark the performance of these systems (Zander et al., 2011; Chi et al., 2012; Liao et al., 2012; Ries et al., 2014). All these investigations reported satisfactory performance of the dry EEG systems/electrodes in comparison to wet systems/electrodes, generally based on the performance of classifiers or the overall average waveform of an event-related potential (ERP). However some authors raised limitations related to timing (Hairston et al., 2014; Ries et al., 2014) and susceptibility to movement artifacts (Chi et al., 2012), which would be particularly problematic in real-world scenarios involving ambulation. Thus, these efforts do not properly encapsulate the challenges likely to be encountered using mobile EEG systems within their target application domain.

Proper benchmarking also requires the selection of metrics that can successfully underpin multiple potential limitations and not simply a comparison of classifier performance. Recent studies focusing on benchmarking EEG systems have been based on a wide range of metrics related to EEG spectral power and amplitude of specific ERP events (Zander et al., 2011; Chi et al., 2012; Ries et al., 2014; Fiedler et al., 2015). However when surveying these studies, a variety of specific metrics are used, highlighting the difficulty of establishing solid methods and metrics for benchmarking. Moreover, studies related to whole-body motion have the addition of substantial movement artifacts (Gwin et al., 2010; Castermans et al., 2014; Reis et al., 2014; Oliveira et al., 2016). Therefore, effective benchmarking for EEG systems towards its usage in whole-body motion conditions is crucial for future advances in mobile brain measurements. In order for benchmarking metrics to be practically informative for real-world applications, they must involve the identification of appropriate metrics for defining the influence of motion on EEG parameters and the reliability of these metrics. The assessment of subject’s comfort and motivation while using EEG systems is another important aspect of the benchmark.

The primary aim of the study described here was to introduce experimental protocols that may serve as benchmarks for assessing the quality of current EEG technologies devoted to mobile recording. Subjects performed an auditory oddball task while seated and during treadmill walking. We calculated basic variables such as absolute power, pre-stimulus noise (PSN), signal-to-noise ratio (SNR) and variance of ERP epochs (CV_ERP_), as well as the level of similarity between ERP curves from seated *vs.* walking conditions. The secondary aim was to investigate the test-retest reliability of these variables. The results of this investigation have fundamental implications on both the usage and community-wide evaluation of mobile EEG systems for exploring brain activity from freely moving humans.

## Materials and Methods

Nine healthy volunteers (6 males and 3 females) between the ages of 21–36 years participated in the study. None had any history of major lower limb injury or known neurological or locomotor deficits. All study procedures were approved by the University of Michigan Internal Review Board and complied with the standards defined in the Declaration of Helsinki. All subjects provided informed, written consent before participating.

### EEG Systems

**Figure 1:**
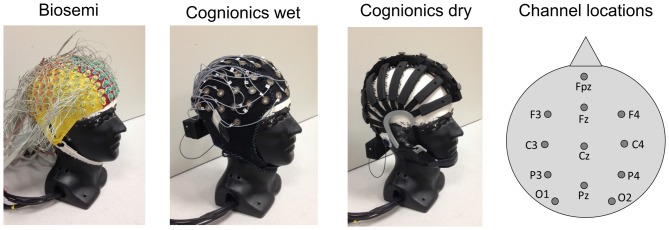
**EEG systems used in the study with locations of the 12 analyzed electrodes**.

All subjects were tested in three different sessions interspaced from 7–40 days. In each session they wore one of the following EEG systems in a random order: (1) Biosemi (248-channel, ActiveTwo, BioSemi, Amsterdam, Netherlands), abbreviated as BSM; (2) Cognionics (72-channel Mobile, Cognionics Inc, San Diego, CA, USA), abbreviated as Cwet; and (3) Cognionics (72-channel Dry, Cognionics Inc, San Diego, CA, USA), abbreviated as Cdry (Figure 1). The Biosemi system uses active electrodes, while both of the Cognionics systems use passive electrodes with active shielding. In each session one of the systems was mounted on the head of the subject following the manufacturers’ recommendations. Subjects were seated in a comfortable, non-reclining chair without any head support or walked at 1.0 m/s on a treadmill while we recorded scalp EEG. For the Biosemi system, gel was used to lower the contact impedance of the electrodes and bring the electrode offset, defined as the running average between the CMS electrode and each of the active electrodes, below 20 mV, as suggested by the manufacturer. For the Cognionics dry system, we made specific adjustments, including moving the electrode legs gently through the hair to touch the scalp and adding small amounts of water at the skin-electrode interface, to optimize the electrode contact with the scalp and bring the impedance for each individual electrode close to 100 kΩ. However, this threshold could not be achieved for all electrodes simultaneously in any data collection. For the wet Cognionics system, we again used gel to lower the contact impedance. We made sure that the impedance of each electrode was below 20 kΩ. Specifically for the Cdry system, after the cap was appropriately positioned on the subject’s head according to the International 10–20 system, each row was lightly tightened so that the electrodes had a firm contact with the scalp without causing any discomfort. The electrodes were also maneuvered through the subject’s hair to ensure they had direct contact with the scalp and were not resting on layers of hair. Subsequently, rows containing electrodes with high impedance were once more gently tightened in order to improve contact between electrodes and the scalp. The total time from cap application to the start of recording was 70 ± 19 min for Biosemi, 38 ± 10 min for Cwet and 28 ± 9 min for Cdry. In evaluating these numbers, please note that the Biosemi system had more than triple the number of electrodes than the two others. In order to assess the test-retest reliability, four subjects could be recruited to perform a second round of tests using the three EEG systems 30–60 days after finishing the first round of tests. This second round of tests were interspaced from 7 to 20 days. These intervals between sessions and for the test-retest intervals were within an acceptable range according to previous work investigating ERP reliability (Lew et al., 2007; Brunner et al., 2013).

### Experimental Protocol

In each experimental session, subjects participated in auditory oddball discrimination and response tasks, once while seated and once while walking. The proposed walking condition was meant to simulate a more realistic movement scenario when compared to the seated recordings. The order of these conditions was randomized across equipment and subjects. Subjects were seated in a padded, non-reclining, stationary chair without head support for the seated portions of the experiment. For the seated portions with the Biosemi system, the A/D box and electrode strands were placed behind their head on a small table so that their weight did not pull backwards on the subject. For the walking conditions, the A/D box was placed in a small backpack and the electrode strands were tethered to the backpack so that the subject had enough slack for a normal range of motion with their head. They were also tethered in such a way that they did not pull the subjects head backwards. The total weight of the backpack they carried was approximately 2.2 kg. There was no need for backpack setup for recordings using Cwet and Cdry systems as they were totally wireless. Subjects were also instructed not to hold onto the treadmill railing unless they needed to do so for their balance. The temperature of the room was set to 72° (F) throughout the experiment. In general, subjects were not visibly sweating and did not report sweating very much when asked. For each condition, participants listened to words presented using E-prime software (Psychology Software Tools, Version 2.0, Sharpsburg, PA, USA) from two speakers placed bilaterally 1.3 m high and 70 cm away from the subjects. Although hearing loss was not objectively assessed, all patients had no difficulty in hearing and responding to the sounds in both conditions. The stimuli were four different color names (blue, red, yellow and green), and one of these four words was selected to be a target (for instance, the word blue) while the other words (red, yellow and green) were non-target. Target and non-target stimuli appeared with a 0.25 and 0.75 probability respectively. Each word sound was presented for 400 ms with a 1420–1580 ms inter-stimulus interval. A fixation point was continuously displayed on a monitor placed at eye level about 1 m in front of the subjects while they were instructed to maintain a fixation on the center of the screen as the stimuli were presented. Subjects were instructed to respond as fast and as accurately as possible by pressing a handheld button every time they heard the target word. If the word was a non-target, they were told not to press the button and wait for the next stimulus. Each participant completed four blocks of 160 trials per condition (seated or walking), and for each block, a different color name was randomly used as target stimulus.

### Recording and Analysis

We recorded EEG for each system on a Windows 7 PC, separate from the E-prime computer, using the native software as provided by the system manufacturers (ActiView, Version 6.05, for BioSemi, and Cognonics Data Acquisition, Version 3.6, for Cognionics). Table 1 shows EEG system specifications (number of channels, sampling rate, signal bandwidth, and reference used). All EEG data were processed in Matlab (The Mathworks, Natick, MA, USA) using EEGLAB and ERPLAB (Delorme and Makeig, 2004; Lopez-Calderon and Luck, 2014). Continuous EEG data from each recorded file were high-pass filtered (1 Hz) and with additional notch filtering using Cleanline.^1^ The use of 1 Hz high-pass filtering might influence the ERP outcomes (Tanner et al., 2015); however for our methodological investigation it became necessary to use this filter for both sitting and walking conditions as described in previous literature (Gramann et al., 2010; Gwin et al., 2010; Ehinger et al., 2014). All datasets from the same condition were concatenated, generating a continuous dataset. Specific frame sequences containing large artifacts resulting from lost packets during wireless telemetry and muscle activity (EMG) were removed from the continuous EEG data. Channels with large fluctuations were marked for rejection and interpolated using EEGLAB. For BSM, we interpolated 1.2 ± 0.4 and 1.4 ± 0.6 channels for seated and walking condition respectively. For Cwet, we interpolated 1.3 ± 0.5 and 1.8 ± 0.5 channels for seated and walking condition respectively. For Cdry, we interpolated 2.1 ± 0.7 and 4.1 ± 1.6 channels for seated and walking condition respectively. Subsequently, independent component analysis was performed on these merged datasets for identifying and removing eye blinks (Ries et al., 2014). In addition, 1–3 components exhibiting large fluctuations in phase with the subject gait cycle were also removed from the walking datasets (Onikura et al., 2015). We identified these components by evaluating the frequency content, which usually has a peak around 1.2 Hz corresponding to the walking frequency. Additionally, we inspected the temporal properties of the IC activation in order to confirm that there was clear periodic oscillation around 1.2 Hz.

**Table 1:** **General specifications for each EEG system**.

EEG system	Sampling rate (Hz)	Bandwidth (Hz)	Reference	# of EEG channels	Wireless transmission	Conductive mechanism
**BSM**	512	0–100	Averaged Mastoids	256	None	Gel
**Cwet**	500	0–131	Linked Mastoid	64	Bluetooth	Gel
**Cdry**	500	0–131	Linked Mastoid	64	Bluetooth	None

The artifact-free datasets were downsized to 12 channels (Fpz, F3, Fz, F4, C3, Cz, C4, P3, Pz, P4, O1 and O2) in order to include samples from each of the primary regions of the cap (De Vos et al., 2014). Preliminary analyses showed these to be sufficiently representative of the primary regions, while also providing common positions across all three systems. Subsequently, these datasets were epoched from −0.3 s to 0.8 s window surrounding the onset of target and non-target stimuli. Non-target stimuli followed by button presses and target stimuli not followed by button presses (i.e., false positives and false negatives, respectively) were rejected. We also rejected epochs based on EEG amplitude thresholds, which were noted for comparison later in the “Results” Section. We evaluated all datasets from seated and walking conditions for each EEG system across a range of amplitude thresholds from 75 to 400 μV. This analysis generated a range of rejection rates used for defining the optimal threshold for further analysis (see “Results” Section for more details). In order to perform fair comparison between seated and walking EEG datasets, we rejected the same number of epochs from both conditions for each system, based on the condition with the most rejected epochs.

From the remaining datasets containing single trials that passed the threshold analysis, we calculated the frequency power (*spectopo* function from EEGLAB, 256 point FFT, 128 point window) for each epoch from each channel separately. Subsequently, we averaged the data from all epochs for each channel and then averaged all channels. This measure, therefore, contains not only the evoked but also the induced power. We calculated the average absolute power across all 12 electrode locations for the, theta (5–8 Hz), alpha (9–13 Hz), beta (13–30 Hz) and gamma bands (30–80 Hz) from both seated and walking conditions. Subsequently we calculated a ratio between EEG absolute power walking/seated (Ratio W/S). Ratios W/S above 1 would indicate that EEG power during walking was higher in comparison to seated. In addition, PSN, defined as the root-mean square of the period from −300 to 0 ms, was calculated for each single trial. The single trial target and non-target datasets were subsequently averaged using ERPLAB and from these averaged curves we calculated the SNR—defined as the ratio of the peak amplitude for P300 and the averaged PSN (De Vos et al., 2014). In addition, we calculated the coefficient of variation—defined as the ratio of the standard deviation to the mean—for trial *i* (*CV(i)*) in a window of 300–500 ms:

$$CV(i)\;=\;\frac{\sqrt{var_{t}\left(EEG(i,t)\right)}}{mean_{t}\left(EEG(i,t)\right)}$$

with *i* as the index of epochs and t as the interval of 300–500 ms. This window contains the P300 event for target stimuli (De Vos et al., 2014). These values were then averaged over epochs (CV_ERP_):

$$CV_{ERP}=\frac{\sum_{i}CV(i)}{n_{i}}$$

with *n*_i_ as the number of epochs. This computation was performed for each channel and each subject in both seated and walking conditions for each system.

In order to quantitatively compare the outcomes of an oddball test during seated *vs.* walking, we calculated the scalar product of target averaged ERPs curves between each subject’s seated and walking conditions for each system separately. Scalar products of normalized vectors yield a score ranging between −1 and +1 representing the overall similarity of a signal (d’Avella et al., 2003) and are equivalent to a correlation coefficient. Thus, scalar products 0.8 > *r* ≥ 0.6 would suggest only moderately similar ERPs recorded for these two conditions, and *r* ≥ 0.8 would suggest highly similar curves for the two conditions. Fisher’s transformation was applied on the individual scalar products values in order to normalize across the distribution for further averaging and back-transformation to the correlation domain.

### Post-Experiment Survey

Immediately after finishing the first session using each of the three headsets, we asked subjects to fill a survey containing the following questions: (1) How well did the cap fit to your head? (2) Did you feel discomfort during the experiment? (3) Did you feel discomfort because the electrodes pinched your head? (4) When did the EEG cap/electrodes start to be uncomfortable? (5) When, if at all, did you lose motivation to continue the experiment? and (6) When, if at all, did you have troubles to stay focused during the experiment? Answers were based on a 5-point Likert scale. No survey was conducted to the four subjects when performing a second round of tests with each system.

### Statistical Analysis

Initially, we performed analysis on EEG epochs based on cumulative distribution function of the single epoch amplitudes, in order to define the amplitude range in which the majority of epochs were found. Subsequently, we applied epoch rejection tool available on ERPLAB based on the range between the minimum and maximum amplitude (200 ms window, 100 ms overlap) in the seated and walking EEG datasets using thresholds varying from 75 to 400 μV. This analysis helped in defining a single threshold for rejecting epochs throughout the subsequent results. After defining the appropriate threshold, we used a 2-way analyses of variance (ANOVA; systems × EEG frequency band) to assess the effects of the EEG systems and EEG frequency band on the Ratio W/S. We also assessed the effects of EEG systems (three systems), condition (seated × walking) and EEG channel (i.e., the 12 electrode locations) on the SNR and CV_ERP_. Due to the exceedingly high rejection rate for the Cdry-walking condition a complete 3-way ANOVA covering all combinations was problematic. Therefore we used two types of 2-way ANOVAS: (1) 2-way ANOVA (3 EEG systems × 12 electrode locations) for the seated condition; and (2) 2-way ANOVA (2 conditions × 12 electrode locations) for Biosemi and Cwet separately. The data from Cdry walking was removed because there was not substantial data available from eight out of nine subjects after thresholding. In case of significant main effects of EEG system and/or EEG channel we performed *post hoc* pairwise *t*-tests with LSD multiple comparison correction.

Log transformation was applied to the variables exhibiting non-normal/skewed distribution. The alpha level of significance was set at *p* < 0.05, and Cohen’s *d* effect size was calculated for all variables (0.2 < *d* < 0.5 = small effect, 0.5 < *d* < 0.8 = medium effect, *d* > 0.8 = large effect). Test-retest reliability was calculated by intraclass correlation coefficient (ICC) method using a two-way mixed effect model set for absolute agreement, as in Brunner et al. (2013) for EEG absolute power in different bands, PSN, SNR, CV_ERP_ and scalar products between seated and walking ERP curves. We used 1-way ANOVA in order to assess the differences among systems for each of the questions in the post-experiment survey, followed by *post hoc* pairwise *t*-tests with LSD multiple comparison corrections.

## Results

### Rejection Rate

**Figure 2:**
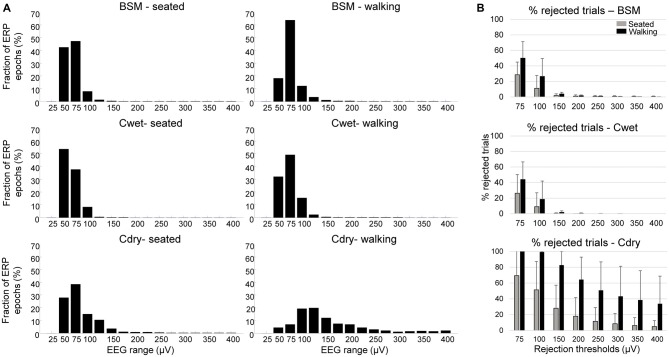
**Fraction of event-related potential (ERP) epochs (%) distributed by signal peak-to-peak amplitude.** In **(A)**, mean distribution of epochs for Biosemi (BSM, *top row*), Cognionics wet (Cwet, *middle row*) and Cognionics dry (Cdry, *bottom row*) in steps of 25 μV (0–25, 26–50, 51–75, …, 376–400) in seated (*left column*) and walking conditions (*central column*). In **(B)**, mean (SD) percentage of rejected ERP epochs for a variety of rejection thresholds in seated (*gray bars*) and walking condition (*black* *bars*).

The average rate at which data must be rejected due to high-amplitude swings, noise, or other artifacts has an impact on the overall usability of the recording. The majority of the ERP epochs showed peak-to-peak amplitudes ranging from 25 to 75 μV for all three EEG systems in seated conditions, as well as for BSM and Cwet in walking conditions (Figure 2A). In the case of Cdry during walking, the majority of the epochs showed peak-to-peak amplitudes ranging from 75 to 125 μV, and ~32% of all epochs showed peak-to-peak amplitudes above 125 μV. Varying the amplitude of cutoff thresholds showed that a traditional threshold at 75 μV may remove ~30% of the seated epochs for BSM and Cwet (Figure 2B), and this same threshold may remove ~68% of the seated epochs for Cdry. There were a higher number of removed epochs for the walking condition regardless the selected threshold. The 75 μV threshold removed 40–50% of the walking epochs for BSM and Cwet, whereas all epochs were marked for removal for Cdry in eight out of nine subjects, and the remaining subject had 94% of all epochs removed. Meanwhile, thresholds above 100 μV selected only a marginal number of walking ERP epochs for rejection for both BSM and Cwet (<4% in average). On the other hand, thresholds at 175 μV and below marked more than 80% of all walking ERP epochs for rejection for Cdry. These differences highlight the challenge associated with recording enough ERP epochs within lower amplitude ranges using Cdry.

### Pre-Stimulus Noise in Different Thresholds

**Figure 3:**
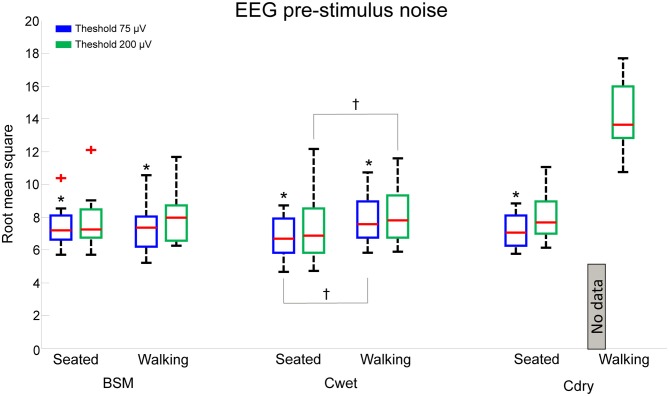
**Boxplots of pre-stimulus noise (PSN) for Biosemi (BSM), Cognionics wet (Cwet) and Cognionics dry (Cdry) for seated and walking conditions at the thresholds of 75 μV (*blue boxes*) and 200 μV (*green boxes*).** No data from Cdry was included since the 75 μV amplitude threshold removed the majority of epochs from walking condition. In each plot the central red mark is the median, the edges of the box the 25th and 75th percentiles. The whiskers cover approx. 99% of the data. *denotes significant difference in relation to 200 μV (*p* < 0.05); ^†^denotes significant difference in relation to walking; ^+^denotes PSN values outside the box limits.

In order to describe the effects of condition (seated *vs.* walking) and different thresholds on a parameter related to EEG data quality, we calculated single epoch PSN by using two thresholds (75 μV and 200 μV; Figure 3). We did not include data from Cdry during walking for the 75 μV threshold as there was no data available from eight out of nine subjects. There were no interaction effects between electrode locations and any other effect (systems or conditions), therefore we averaged the 12 electrode location values for each subjects for further display in Figure 3. The separate ANOVAs for each system revealed a main effect of threshold on PSN results. This specific analysis did not aim at comparing performance across systems, but rather targeted the performance of a given system in two conditions (seated *vs.* walking) and two thresholds (75 *vs.* 200 μV) in order to define whether the threshold is a critical factor in determining the quality of the acquired EEG epochs. There was a significantly lower PSN for the 75 μV threshold in comparison to 200 μV for BSM (*F*_(1,96)_ = 5.62, *p* < 0.05), Cwet (*F*_(1,96)_ = 6.01, *p* < 0.05) and Cdry (*F*_(1,96)_ = 34.3, *p* < 0.001). In addition, there was a main effect of condition for Cwet (*F*_(1,96)_ = 12.2, *p* < 0.001, effect size = 0.7), in which lower PSN was found for the seated condition in comparison to walking. These results suggested that there were no main effects of condition for the PSN recorded from BSM for both thresholds of 75 μV and 200 μV. Therefore this system performs equally in seated and motion conditions. Cdry showed substantial influence of motion, which made inviable the use of data from 75 μV threshold in walking conditions, and PSN from 200 μV threshold was substantially higher in comparison to those from wet systems. Based on these results, we fixed our threshold at 75 μV for the additional analysis presented in the following results, and there were no results from Cdry in the walking condition, since this threshold removed all epochs for eight out of nine subjects.

### Spectral Analysis

**Figure 4:**
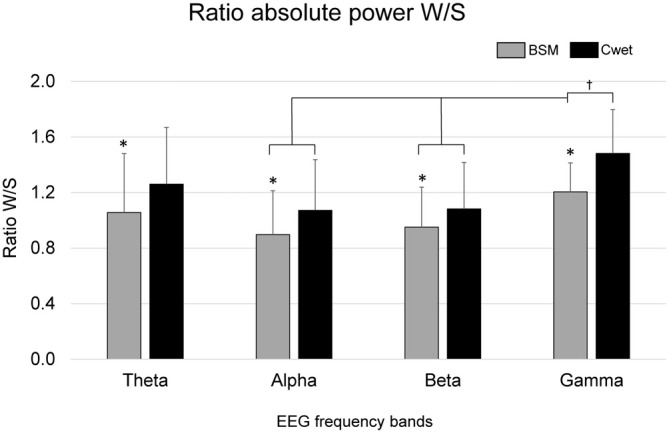
**Mean (SD) ratio of absolute power walking/seated (Ratio W/S) for Biosemi (BSM) and Cognionics wet (Cwet) in the Theta (4–8 Hz), Alpha (8–13 Hz), Beta (13–30 Hz) and Gamma (30–80 Hz) frequency bands.** No data from Cdry was included since the 75 μV amplitude threshold removed all epochs from walking condition; *denotes significant difference in relation to Cwet (*p* < 0.05); ^†^denotes significant difference in relation to the indicated frequency bands (*p* < 0.05).

In order to ascertain differences in sensitivity to spectral power in seated and walking conditions, we calculated the Ratio W/S. Cdry has been excluded from this analysis due to the lack of data from walking condition. A 2-way ANOVA (2 EEG systems *vs.* 5 EEG frequency bands) was used and a main effect of EEG system was found (*F*_(1,64)_ = 6.73, *p* < 0.05). The Ratio W/S for BSM was significantly lower in comparison to Cwet across all frequency bands (Figure 4). In addition, there was a main effect of EEG frequency band on the Ratio W/S (*F*_(3,64)_ = 4.64, *p* < 0.01). The ratio W/S from the delta band was significantly higher than theta and alpha bands (*p* < 0.01, effect sizes between 0.5 and 0.7), and the ratio from gamma band was significantly higher than alpha band (*p* < 0.01, effect size = 0.75) and beta band (*p* < 0.01, effect size = 0.82). The result from the ratio W/S show that BSM can provide ratios closer to 1, indicating better quality of results from walking recordings in comparison to Cwet. Moreover, we found consistent differences across EEG frequency bands between BSM and Cwet.

### Signal-to-Noise Ratio

**Figure 5:**
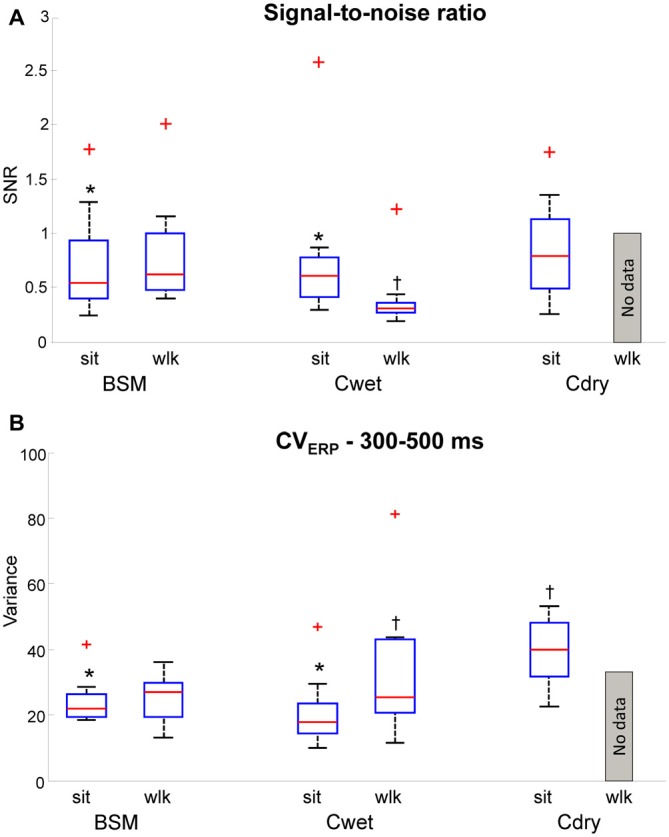
**Boxplots representing signal-to-noise ratio (SNR; A, *upper panel*) and EEG variance (CV_ERP_) across 300–500 ms after stimulus presentation (B, *lower panel*) in seated (sit) and walking conditions (wlk) for the three EEG systems used (Biosemi: BSM, Cognionics wet (Cwet) and Cognionics dry (Cdry)).** For each panel, data from all 12 electrode locations were included in the boxplots. No data from Cdry was included since the 75 μV amplitude threshold removed most of the epochs for this system and condition. In each plot the central red mark is the median, the edges of the box the 25th and 75th percentiles. The whiskers cover approx. 99% of the data. *denotes significant difference in relation to walking condition for the same system. ^†^denotes significant difference in relation to seated condition from all EEG systems; ^+^denotes SNR values outside the box limits.

The SNR is a variable widely used to describe the quality of EEG epochs with respect to the background noise inherent to EEG acquisition. We calculated SNR for each of the 12 channels for all EEG systems in the seated condition and from BSM and Cwet during walking. We investigated the effects of electrode location and condition for BSM and Cwet separately, using 2-way ANOVAs. In addition, we investigated the effects of electrode location and EEG system for the seated and walking conditions separately. For the seated condition we computed differences between BSM *vs.* Cwet *vs.* Cdry, whereas for walking we just computed differences between BSM *vs.* Cwet. There was no interaction between electrode location and condition, as well as no interaction between electrode location and systems (*p* > 0.05). There was interaction between EEG systems and condition for the SNR (*F*_(1,192)_ = 8.70, *p* < 0.01), showing that Cwet in walking had significantly lower values in comparison to BSM in the seated condition (Figure 5A). In addition, there was a significant main effect of EEG system (*F*_(2,192)_ = 11.94, *p* < 0.001). *Post hoc* *t*-tests with LSD multiple comparison correction revealed that Cwet had the highest SNR in the seated condition, followed by BSM. We also found a significant main effect of condition (*F*_(1,96)_ = 21.98, *p* < 0.001), in which the SNR in the seated condition was significantly higher than walking for Cwet. The results from SNR indicated that all EEG systems have similar performances in the seated condition. However recording EEG data during walking reduces the quality of the signals for Cwet, whereas BSM can actually show increased SNR.

### EEG Amplitude Variance

The variable CV_ERP_ was calculated in order to quantify the variability inherent to a window containing the P300 peak for individual epochs. Higher variances mean potential changes in the amplitude pattern that can influence the ERP results. For the purpose of this analysis we assume that CV_ERP_ might be higher during walking due to motion artifacts. Yet, high-quality systems must allow recordings with minimal differences between seated and walking conditions. The statistical design was the same as applied for SNR. The results revealed interaction between EEG system and condition (*F*_(1,192)_ = 55.78, *p* < 0.001), showing that Cdry in seated condition had significantly higher CV_ERP_ in comparison to other EEG systems in the same condition (Figure 5B, data from all channels combined in the boxplots). In addition, we also found a significant main effect of condition (*F*_(1,96)_ = 42.21, *p* < 0.001), in which CV_ERP_ in seated condition was significantly higher than walking for BSM. No significant main effect of electrode location was found (*p* > 0.05). Our results from CV_ERP_ demonstrated that, similarly to the SNR, BSM can maintain the same level of quality on the data recorded during walking, whereas Cwet showed increases in variance in walking conditions. Moreover, Cdry delivered data from seated condition that was more variable than all datasets from wet systems.

### Similarities Seated *vs.* Walking

**Figure 6:**
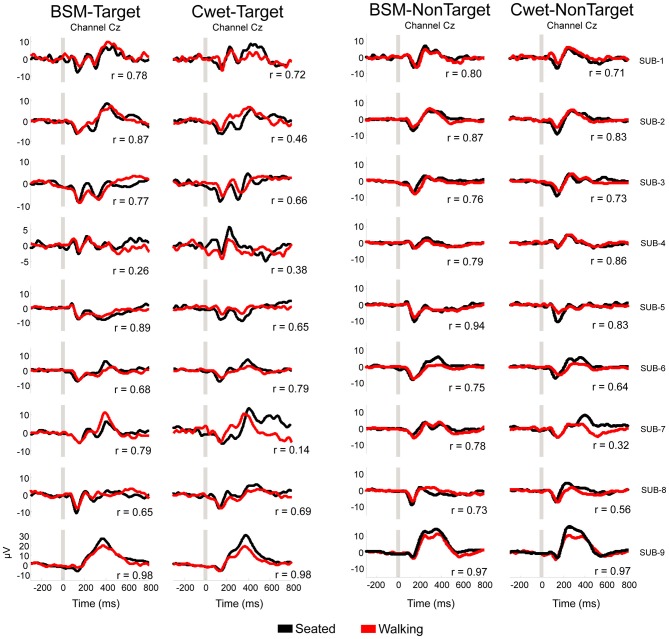
**Averaged target and non-target curves from auditory ERP during seated (*black traces*) and walking (*red traces*) for each subject plotted in different rows (SUB-1 to SUB-9), and different EEG systems in the columns.** For each pair of curves, *r* is the scalar product of the comparison between these curves. No data from Cdry was included since the 75 μV amplitude threshold removed all epochs from walking condition.

**Figure 7:**
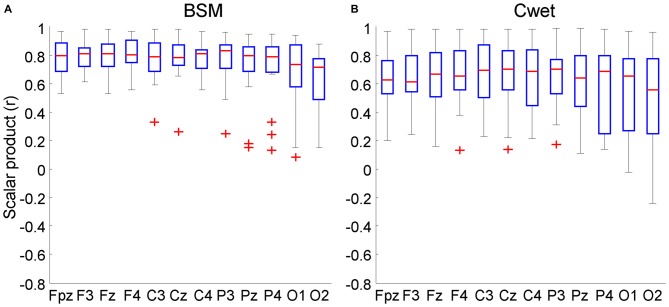
**Boxplots of the scalar products of the comparison seated *vs.* walking for both target and non-target conditions combined for each of the 12 electrode locations.** EEG data was recorded from Biosemi (BSM, **A**) and Cognionics wet (Cwet, **B**). No data from Cdry was included since the 75 μV amplitude threshold removed all epochs from walking condition. In each plot the central red mark is the median, the edges of the box the 25th and 75th percentiles. The whiskers cover approx. 99% of the data. ^+^denotes samples outside the boxplot limits.

Scalar products provide an objective measurement for the overall similarity of signals without regard to potential differences in scaling, and a means to assess any distortion observed between seated and walking ERP curves. The comparison between parameters such as P300 peak extracted from seated and walking conditions show similar results using traditional ERP methods (Gramann et al., 2010; Debener et al., 2012). However, it is possible that ERP epochs may be affected by motion artifacts during recordings while walking. The scalar products were included in this analysis in order to provide quantitative comparison of the entire ERP waveforms between conditions. Illustrations of target ERP curves from the sitting and walking conditions for both wet EEG systems are shown in Figure 6 for the target and non-target stimuli from the Cz electrode. In general, BSM and Cwet presented predominantly similar patterns in the two conditions (5 out of 9 subjects with *r* > 0.75). Regarding all analyzed channels, the grand average across all channels (including target and non-target stimuli) for BSM was *r* = 0.75 ± 0.18. There were 186 scalar products (>80% of the total from 9 subjects × 12 electrode locations × target/non-target stimuli) showing moderate or high similarity (*r* > 0.6). However, channels located posteriorly in the head (O1, O2) show reduced similarity (Figure 7A). For Cwet, the grand average was *r* = 0.62 ± 0.25 and also follows the same trend of reduced similarities for channels located posteriorly in the head (Figure 7B). There were 134 scalar products (>60% of total) showing moderate or high similarity. The results from scalar products confirmed the superior performance of BSM and lower variability of observed event related potentials in comparison to Cwet. Moreover, both systems demonstrated limitations in delivering similar ERP curves from EEG electrodes located posteriorly in the head.

### Test-Retest Reliability

**Figure 8:**
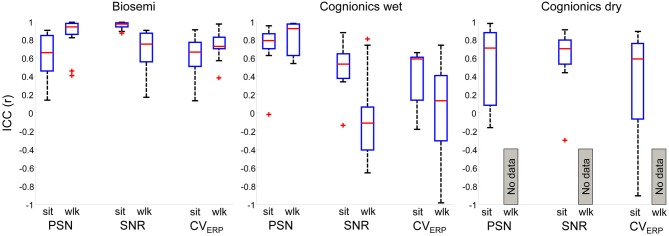
**Boxplots representing Intra class correlation coefficient (ICC) of 12 electrode locations for PSN, SNR and EEG variance (CV_ERP_) in seated (sit) and walking (wlk) conditions.** Data was recorded from Biosemi (*left* *panel*), Cognionics wet (*central panel*) and Cognionics dry (*right panel*). No data from Cdry walking was included as the 75 μV amplitude threshold removed all epochs for this system and condition. For each panel, data from all 12 electrode locations were included in the boxplots. In each plot the central red mark is the median, the edges of the box the 25th and 75th percentiles. The whiskers cover approx. 99% of the data. ^+^denotes ICC values outside the box limits.

In order to assess overall consistency of the different metrics above across the recorded signal, test-retest reliability was calculated by ICC. That is, we compared the characteristic derived parameters PSN and CV_ERP_ in different conditions between the first test and the re-test. This does not imply a direct comparison of an ERP waveform. We have found different results depending on the EEG system. BSM showed good median reliability (ICC > 0.70) for all variables across all 12 electrode locations. For PSN and CV_ERP_ the ICC was slightly higher for walking in comparison to the seated condition, whereas the ICC was higher for SNR in the seated condition (Figure 8). The ICC for BSM presented lower variability in comparison to the two other systems Therefore, data collected using this system can be considered better reproducible in seated and walking conditions.

The results for Cwet were less consistent. The PSN showed good reliability for both seated and walking conditions, however for both SNR and CV_ERP_ the ICC was reduced. The ICC for SNR was moderate for the seated condition, but for walking it was poor (ICC < 0.4). The same was found for CV_ERP_. Moreover, the ICC for walking showed large variability for SNR and CV_ERP_. Thus, the use of Cwet can provide reproducible results depending on the variable, and also if data is recorded in stationary conditions.

Cdry showed moderate to good median reliability in all three variables in the seated condition, although the variability was high for PSN and CV_ERP_. The SNR was the most stable variable while showing low variability. We could not evaluate the performance of this system during walking as the data could not be used for the calculation of such variables.

The ICC calculated from the EEG absolute power was predominantly moderate/good for all EEG systems in seated and walking conditions (Table 2). Exception was found only for the theta band for Cdry seated condition (ICC < 0.5). These results suggest that the absolute power is also a reproducible variable from EEG recordings in seated and walking conditions.

**Table 2:** **Intra class correlation coefficient (ICC) of EEG absolute power in seated (sit) and walking (wlk) conditions for each EEG frequency band**.

	Intra-class correlation coefficient (ICC) – Absolute power			
	Theta	Alpha	Beta	Gamma
*Seated*
BSM	0.63	0.99	0.99	0.49
Cwet	0.76	0.84	0.93	0.68
Cdry	0.47	0.84	0.86	0.82
*Walking*
BSM	0.96	0.98	0.98	0.98
Cwet	0.93	0.99	0.99	0.95

*Data was recorded from three EEG systems (Biosemi: BSM, Cognionics wet (Cwet) and Cognionics dry (Cdry)). No data from Cdry was included in the walking condition since the 75 μV amplitude threshold removed all epochs for this system and condition*.

### Post-Experiment Survey

**Figure 9:**
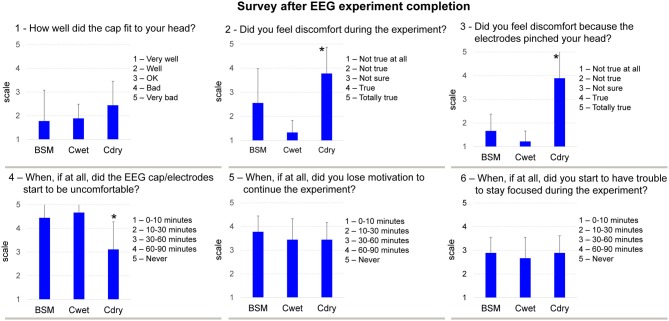
**Mean (SD) scale from the post-experiment survey.** For each question the scale varies as stated in the subpanels. *denotes significant difference in relation to BSM and Cwet (*p* < 0.05).

After completing the experiment, most of the subjects reported that the three different EEG systems fit their heads well/very well for the wet systems and well/OK for the dry system (Figure 9, question 1), with no significant differences across systems. For questions 2 and 3, subjects reported significantly higher discomfort using Cdry (*F*_(2,24)_ = 11.62, *p* < 0.001), and this discomfort was caused by the electrodes pinching their heads during the experiment (*F*_(2,24)_ = 26.81, *p* < 0.001) in comparison to the other two wet systems. Moreover, in question 4, subjects reported that they started feeling discomfort using Cdry approximately 30–60 min through the experiment, which was a significantly shorter time in comparison to the other two EEG systems (*F*_(2,24)_ = 5.63, *p* < 0.05). No significant differences were found in relation to the time subjects started to lose motivation (question 5), but there was a trend to extended motivation while using BSM. In addition, no significant differences across EEG systems were found in relation to the time subjects started to lose focus (question 6).

## Discussion

**Figure 10:**
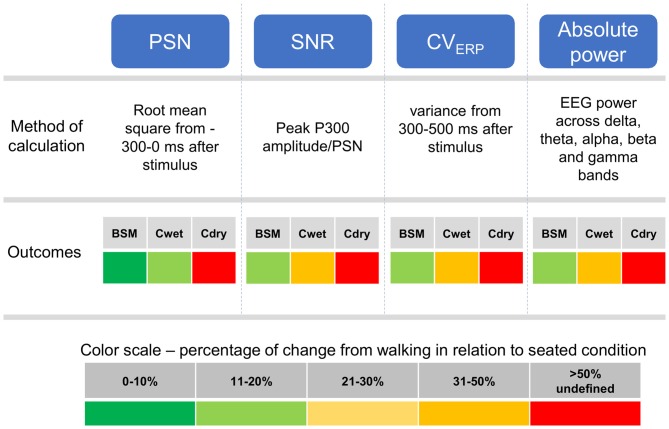
**Description of the methods of calculation for PSN, SNR, EEG variance (CV_ERP_) and absolute power in the five EEG frequency bands.** The fields outcomes describe the absolute average percentage of change across all subjects between seated and walking conditions in a color scale for Biosemi (BSM), Cognionics wet (Cwet) and Cognionics dry (Cdry). No data from Cdry walking was included as the 75 μV amplitude threshold removed all epochs for this system and condition, therefore the comparisons for Cdry were marked as undefined.

Scalp EEG is currently the main tool for studying brain activity in real-world conditions. There is an increasing number of EEG systems suitable for recordings during whole-body motion, but there is an overall lack of community agreed-upon benchmarks for defining the performance and usability of such EEG systems. In this study, we proposed a standard auditory oddball task and basic metrics that were effective in underpinning differences in the performance and usability of three EEG systems. We first found that EEG epochs from the dry EEG system had substantially higher peak-to-peak amplitudes for both seated and walking conditions. Furthermore, data from the walking condition was mostly unusable due to high rejection rate when using a typical maximum threshold value. Secondly, calculations of PSN, SNR, CV_ERP_ and scalar products revealed differences across systems and/or across conditions (seated *vs.* walking). These variables showed predominantly moderate/good reliability, especially for the seated condition. Finally, subjects felt less discomfort and were motivated for longer periods while using wet EEG systems in comparison to the dry system. These results combined suggest that the proposed methodology and subsequent analysis were successful in identifying differences across systems that are mostly caused by motion-related artifacts and usability issues. A short description of the computation of proposed variables and a brief summary of the results from the comparison between seated and walking for each EEG system is shown in Figure 10.

Previous investigations have applied a threshold of 75 μV for cognitive tasks (De Vos et al., 2014; Ries et al., 2014). However, we found that different EEG systems may have widely different numbers of rejected epochs at such specific amplitude thresholds. Although BSM and Cwet had acceptable numbers of rejected epochs (about 15–40% rejection rate), the higher rejection rate for Cdry indicates an inherent source of noise, especially during walking. The use of the usual threshold determined that it was not possible to keep data from Cdry during walking throughout the entire investigation. There was an unexpectedly high rejection rate for both wet systems in the seated condition (~30%), which may be related to the use of 12 electrodes from different head locations containing artifacts not removed by the ICA cleaning. For both wet systems, there was an expected increase in the rejection rate in walking conditions, and both systems showed similar performance. Thus, we suggest that the use of standard amplitude thresholds for defining the rejection rate was successful in discriminating the performance of EEG systems. We speculate that the differences between seated and walking conditions for Cdry can be explained by the higher susceptibility for changes in contact impedance of the dry electrodes from head motion caused by walking dynamics. Furthermore, a noted difficulty in establishing optimal levels of impedance across all rows of electrodes during subject preparation before the experiment can also be directly related to these high rejection rates. We based our ERP results on the averaging of a similar number of epochs across systems and conditions, and in some subjects we rejected ~50% of all trials’ target epochs due to the high rejection rate for Cdry. Previous studies have recommended the average of at least 70 trials for optimal results concerning P300 (Kiesel et al., 2008). Our results follow this recommendation, as the rejection of 50% of target epochs would still result in averaging 80 epochs.

We calculated PSN as a benchmark metric since it can quantify the signal quality (De Vos et al., 2014). Ideally this amplitude fluctuation should be robust across seated and walking conditions, since the motion-related artifacts and the stimulus presentation were not coupled. Although three EEG systems showed similar PSN in the seated condition, only BSM did not show altered values while comparing seated *vs.* walking. A recent investigation did not find differences in PSN recorded in seated and walking conditions using an adapted Emotiv headset (De Vos et al., 2014). However, there were differences in the methods used in this investigation and our results. De Vos et al. (2014) reported the PSN only for channel Pz, as representative of all other selected channels, whereas we used all 12 electrode locations in order to assure a robust representation of the EEG data across the scalp. Nonetheless, the use of PSN was effective in discriminating the performance of EEG systems when comparing seated *vs.* walking conditions.

The ratio W/S showed that different EEG frequency bands can have distinct influences of motion-related artifacts for both wet systems. In addition, the greater ratio W/S for Cwet suggests that power spectrum for this system is more influenced in walking conditions, when compared to BSM. The ratio W/S has the advantage of excluding normalization issues, since the ratio is calculated within the seated and walking EEG power for the same system. Previous investigations showed predominantly similar power spectrum for EEG recordings from wet and dry EEG electrodes in static (Zander et al., 2011; Chi et al., 2012) and dynamic conditions (Lin et al., 2014). However, these studies were conducted using different types of electrodes in simultaneous recordings. Our results for benchmarking focused on comparing entire systems, which can explain potential differences to previous research. As suggested in the “Results” Section, the ratio W/S from specific EEG epochs should ideally be as close to 1 as possible, if there are no major changes in continuous electrocortical activity between seated and walking conditions. Previous studies have shown reduction in alpha and beta power during motor activity (Tzagarakis et al., 2015), which can explain the ratios W/S below 1 for Biosemi for these two frequency bands. However, ratios from Cwet were consistently above 1, suggesting that higher power during walking recordings in the alpha and beta bands may be due to movement artifacts. Although the difference in the ratio W/S is not substantial across systems, this metric was effective in determining changes in the EEG frequency content related to motion.

The SNR was greater for BSM in comparison to all other EEG systems, and the CV_ERP_ for BSM was lower in comparison to all other EEG systems. The SNR is a traditional metric for quantifying the quality of the target ERP event in relation to the baseline noise. EEG is well-known for not presenting optimal SNR (Reis et al., 2014), and ERP events such as the P300 might be highly influenced by changes in EEG amplitude. It is noteworthy to mention that a few subjects have not presented clear P300 events (see subjects 4 and 5 in Figure 6) as previously reported elsewhere (Picton, 1992; Polikar et al., 2007). These subjects did not present clear P300 events regardless of the EEG system or condition, highlighting the importance of standardized benchmarking procedures for comparing the performance of EEG technologies. The use of SNR as a benchmark metric showed that motion did not negatively influence the SNR for BSM, whereas it was reduced for Cwet. The reduction in SNR for Cwet during walking can be explained by the greater PSN found in this condition. On the other hand, SNR can be also influenced by the amplitude of the P300 event. We calculated CV_ERP_ in order to quantify the influence of motion on the selected ERP epochs, even though all epochs passed the EEG amplitude rejection method. CV_ERP_ showed the increase in trial-by-trial variance in walking conditions for both wet systems, especially for Cwet. Our results suggest that the use of SNR alone as a metric for benchmarking may generate questionable results, but the combination of SNR and CV_ERP_ can establish a robust method for evaluating the variability across individual epochs for different EEG systems. This suggestion can be supported by the results from Cdry while seated, as we found a high SNR, but there was also a high CV_ERP_ in the P300 window. The Cdry system showed to be very sensitive to motion, and minimal head motion can add artifacts to the acquired EEG data. Moreover, there might be other sources of artifacts related to dry electrodes not studied in our work. There is an inherent higher variability in the epochs for Cdry, as measured by the CV_ERP_. Therefore it is not possible to assure that higher SNR is directly related to higher overall signal quality.

Previous studies comparing event-locked EEG data from stationary and dynamic conditions have used metrics such as peak P300 and PSN (Gramann et al., 2010; Debener et al., 2012) calculated from averaged ERP epochs. Our study introduced a comparison of the ERP curves from seated and walking conditions by means of scalar products. This methodology is equivalent to computing the correlation over time and allows for a direct mathematical comparison of the averaged epochs from both conditions, complementing the results already explored in the literature. Previous studies applied correlation analysis to investigate how similar power spectral curves from wet and dry electrodes were shaped, showing that both types of electrodes have similar spectral distribution (Zander et al., 2011; De Sanctis et al., 2014). Ideally, target and non-target curves from sitting and walking should be as similar as possible in a controlled laboratory-based setup, since no distraction other than the treadmill movement and its inherent noise are added while walking. This hypothesis is confirmed by the fact that more than 80% of all comparisons for BSM showed scalar products above 0.6 and out of these 40% being highly similar. Similar analyses for Cwet, showed poorer performance when compared to BSM, suggesting changes in the ERP curve shapes related to whole-body motion. Furthermore, channels located in the frontal and central areas of the head presented higher similarity to the target curves in comparison to those located in the posterior area (Figures 7A,B). These results suggest that the evaluation of the entire ERP curve can be used for establishing overall signal quality between different recording conditions.

In addition to identifying appropriate metrics for benchmarking EEG systems, we tested the potential reliability of such metrics. Overall, a general statement of which are the best metrics in terms of reliability is difficult to achieve, as different systems demonstrated different performances in each variable. Nonetheless, BSM continued to show moderate/good ICCs for all variables in both seated and walking conditions, whereas Cwet showed reduction in the reliability for SNR and CV_ERP_, especially in walking conditions. Previous investigations have described good test-retest reliability of evoked EEG responses from transcranial magnetic stimulation (Lioumis et al., 2009), and auditory responses using ICC (Brunner et al., 2013) and Pearson correlation (McFadden et al., 2014). All these previous investigations were conducted in seated conditions, and to our knowledge, the present investigation is the first to report test-retest reliability for EEG experiments in dynamic conditions. Although there was a limited sample size for test-retest reliability, our robust results suggest that wet systems deliver reliable results between sessions in seated conditions, but reliability is reduced in walking conditions.

Our results from the post-experiment survey showed that subjects felt discomfort while using Cdry, and this discomfort was caused by the electrode pressing against the scalp and pinching their heads. In fact, five subjects reported headaches after the experimental session, which can be related to the pressure of the dry electrodes onto the head. EEG experiments have shown that headache and discomfort can cause a reduction in cognitive performance (Evers et al., 1997; Lorenz and Bromm, 1997). These results are essential for EEG system developers because discomfort can limit cognitive performance and change the expected electrocortical signals in real-world conditions. The comfort rating of the Cdry in our experiment does not corroborate previous investigations that reported good comfort rates for dry EEG technologies (Hairston, 2012; Chen et al., 2014; Fiedler et al., 2015). However, these investigations were assessing comfort using different scales, from different number of electrodes and EEG sensors. The cap placement and preparation for the wet systems demanded more time in comparison to the dry system. Nonetheless, subjects did not report problems regarding the long procedure. Further studies and standardization of methods for accessing comfort are needed in order to establish optimal methodologies for benchmarking the usability of EEG systems. Additionally, our study has a limitation concerning number of subjects, especially for the test-retest reliability. We found robust ICC especially for the wet systems, which suggests that the proposed metrics are reliable. However future studies aiming at developing these benchmarking testing protocols should consider using larger subject populations to gather more robust information about the different methodologies.

In summary, we propose the auditory oddball paradigm in combination with the calculations of epoch rejection rate, PSN, SNR, CV_ERP_ and scalar products can be used as a benchmark method for testing the performance of different EEG systems. Furthermore, we observed that a walking *vs.* seated paradigm elicits strong differences in these metrics. The use of wet systems has demonstrated the potential of acquiring EEG data with comparable quality in seated and walking conditions. The investigated metrics were successful in underpinning which systems were suitable to deliver comparable EEG data in whole-body motion, and the robustness of such results were attested by the assessment of test-retest reliability. Finally, EEG systems based on dry electrodes may need substantial improvement to meet the quality standards of wet electrodes. At the same time, improvements on the electrode construction and materials is needed, since comfort and performance may be compromised.

## Author Contributions

ASO, BRS, WDH, PK and DPF designed the experiment. ASO and BRS performed the experiments. ASO, BRS, WDH, PK and DPF analyzed and interpreted the data. ASO, BRS, and DPF drafted the manuscript and all authors approved the final version.

## Funding

This work was supported in part by the US Army Research Laboratory ARL-CTA (W911NF-09-1-0139 and W911NF-10-2-0022).

## Conflict of Interest Statement

The authors declare that the research was conducted in the absence of any commercial or financial relationships that could be construed as a potential conflict of interest.

## Footnotes

^1^https://www.nitrc.org/projects/cleanline/
